# Numerical Network Modeling of Heat and Moisture Transfer through Capillary-Porous Building Materials

**DOI:** 10.3390/ma14081819

**Published:** 2021-04-07

**Authors:** Borys Basok, Borys Davydenko, Anatoliy M. Pavlenko

**Affiliations:** 1 Head of the Department of Thermophysical Basics of Energy-Saving Technologies, Institute of Engineering Thermophysics National Academy of Sciences of Ukraine, 03057 Kyiv, Ukraine; basok@ittf.kiev.ua (B.B.); bdavydenko@ukr.net (B.D.); 2 Department of Building Physics and Renewable Energy, Kielce University of Technology, al. Tysiąclecia Państwa Polskiego 7, 25-314 Kielce, Poland

**Keywords:** porous medium, heat transfer, mass transfer, mathematical modeling, numerical research methods

## Abstract

The article presents the modeling of the dynamics of the vapor-gas mixture and heat and mass transfer (sorption-desorption) in the capillary structure of the porous medium. This approach is underpinned by the fact that the porous structure is represented by a system of linear microchannels oriented along the axes of a three-dimensional coordinate system. The equivalent diameter of these channels corresponds to the average pore diameter, and the ratio of the total pore volume to the volume of the entire porous material corresponds to its porosity. The entire channel area is modeled by a set of cubic elements with a certain humidity, moisture content, pressure and temperature. A simulation is carried out taking into account the difference in temperatures of each of the phases: solid, liquid and gas.

## 1. Introduction

Most of the materials used in construction have a capillary-porous structure. The thermal insulation properties of these materials depend on the condition parameters: temperature, pressure, humidity and moisture content. Predicting the heat loss levels from the premises to the surrounding space through enclosing structures depends on the accuracy and reliability of heat and mass transfer through the capillary-porous media.

Many computational schemes use models based on the phenomenological theory of mass and heat transfer [1,2,3], whereby a real porous structure is replaced by a homogeneous continuous medium. The transfer processes for this continuous medium are expressed by mass and energy conservation equations, where volume-averaged physical values and effective transfer coefficients are used [4,5,6,7].

**Figure 1:**
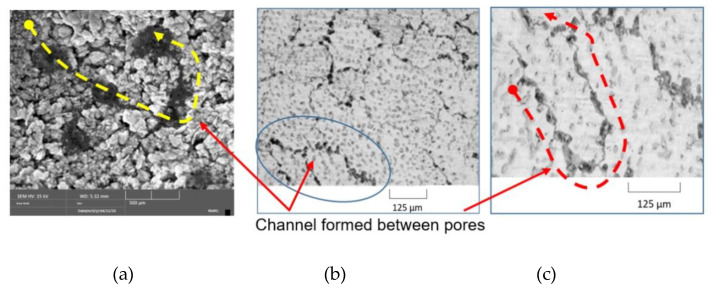
Structure of materials: (**a**) granular concrete; (**b**), (**c**) cellular concrete.

This approach is quite justified, as the shape of pores, their quantity and distribution in the material volume are random parameters, if we do not mean formed cracks in pore connecting interpore space or channel porosity. The shape of such pores has a pronounced configuration and size. It is the channel porosity (cracks, as shown in Figure 1) that can significantly change thermophysical properties of the material. Naturally, in this case, averaging of physical values over material volume results in errors in the calculations of heat and mass transfer parameters.

In some cases, the use of this approach to solving problems of heat and mass transfer results in uncertain individual values of transfer equations. In particular, it refers to source terms, included with different signs in liquid and vaporous moisture mass conservation, and expressing the moisture transition rate from one phase to another, during liquid evaporation or condensation inside the material.

As it is difficult to determine this value, both mass conservation equations are usually summed up. The resulting mass transfer equation no longer contains the specified value. However, in this case, the resulting equation describes the transfer of a certain total moisture content, including both liquid and vapor phases. In this instance, the moisture evaporation or condensation rate inside the material remains in the energy equation. Many researchers use this technique. But at the same time the physics of the effects of evaporation (condensation) remain undisclosed. We, however, avoided the indicated method and directly considered the effects of the phase transition-evaporation or condensation. This is the main idea of the article.

For example, this approach is applied in the work [3], where the authors propose a one-dimensional model, consisting of energy, dry air and total humidity equations.

In the work [5], they propose a mathematical model for the drying of wet building materials, taking into account the presence of water and vapor. Pressure and temperature are taken as variables. The authors consider simultaneous capillary water transfer and vapor diffusion in two-dimensional areas. The effect of dry air movement was not considered in these models. In the work [8], a mathematical model is represented by equations of moisture and heat, transferred through a silica brick; these parameters were taken as independent variables. In the work [9] the same approach is proposed, but moisture and heat are transferred through a complex anisotropic material structure. In the presented works, the models take into account three basic phenomena: vapor diffusion, capillary suction in a porous medium and advective transfer of moist air through thin channels. A similar calculation scheme for moisture transfer in brick is presented in [10] and it is based on the same control potentials.

An expanded mathematical model of heat and mass transfer in the homogeneous porous building materials is presented in [11,12,13]. It includes four basic transfer equations: water vapor, dry air, liquid moisture and energy. Dry air and water vapor densities, as well as a volume fraction of liquid moisture and temperature, are used as independent variables. The analyzed building material, namely brick, is considered as a porous material. A solid phase is the material from which the brick is made; water and moist air are present in its pores. The amount of water in the building material pores changes as a result of the transfer caused by capillary pressure gradient, as well as evaporation and condensation processes, while the amount of vapor also changes as a result of diffusion and phase transition processes. In the presented models, phase heat equilibrium is assumed, therefore a unified equation of energy transfer is considered. It also assumes averaging the parameters within material volume.

Another approach, used to describe heat and mass transfer processes in capillary-porous materials, is associated with a model of the evaporation zone deepening [14,15,16]. According to this model, there are dry and moist zones in a wet material. In a dry zone, moisture is present only in a gaseous form (as vapor), and in the moist zone, all pores are occupied by liquid moisture. The liquid evaporates only at the interface of these zones, it is deepening towards the wet moist zone. It is assumed that the heat is supplied to the evaporation boundary by applying thermal conductivity of the material dry layer and spent on moisture evaporation.

The mathematical formulation of this process is based on a Stefan-type problem [17]. Similar models are proposed in the works [14,15]; however, they neither consider radiation heat transfer on the dried surface, nor analyze the step size sensitivity or computational grid density. Currently, the heat and mass transfer models, based on the capillary-porous structure, represented as the so-called pore network, are used [18,19,20,21,22,23]. According to this model, a real microstructure of the porous material is replaced by a system of interconnected and intersecting channels with a known arrangement and geometric dimensions. Results of the mass transfer study, using this approach, are presented in [24,25,26,27,28,29,30].

**Figure 2:**
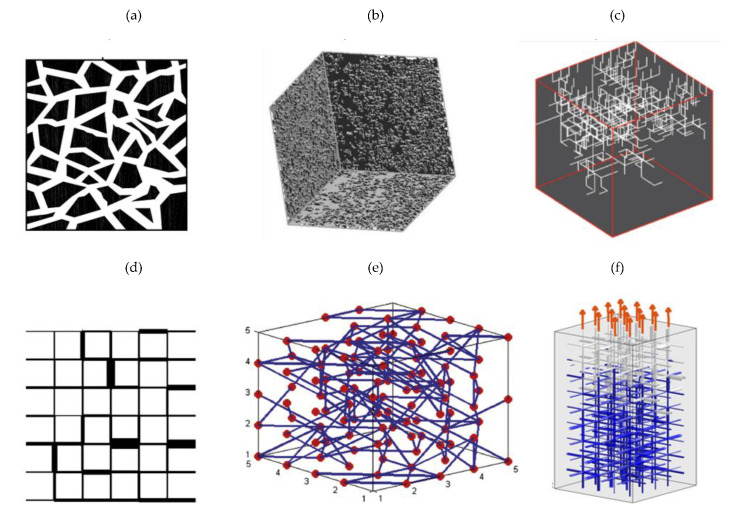
Network models: (**a**)–[4]; (**b**)–[5]; (**c**)–[6]; (**d**)–[7]; (**e**)–[8]; (**f**)–[9].

Figure 2 shows the most common network models, where pores are represented by lines.

In these works, several numerical approaches are proposed for modeling the transfer of heat, mass and momentum during porous material dehydration. These approaches are characterized by spatial scale and physical processes to be reflected in the models. These models consider the material as a continuum divided into microvolumes. It is assumed that in these microvolumes (MV) individual phases are superimposed on each other, meaning that they cannot be analyzed separately. Therefore, MV should be large enough, for example larger than the pore size, in order to provide averaging of material properties within the MV. On the other hand, MV should also be small enough to prevent changes in the studied parameters within these volumes (e.g., temperature), resulting from macroscopic gradients and associated nonequilibrium conditions at this microscale level. Transfer inside the material is modeled by averaged material properties, obtained either experimentally, or by numerical calculation. Thus, complex pathways and microscale transfer processes are included in a concentrated way in the material properties and transfer equations, instead of explicitly taking them into account by modeling. A typical example is the use of the Darcy’s law combined with fluid permeability, i.e., a macroscopic material property, in order to describe the fluid transfer inside a porous material at the continuum level, inherently including complex transfer phenomena at the microscale level. These material properties are often a complex function of temperature and moisture.

In the works [22,23], it is shown that the model of a porous medium drying zone is the result of generalization of many phenomenological observations and experimental studies, and describes liquid phase distribution during drying of porous media. But they fail to explain the internal mechanism of the “evaporation zone” phenomenon. Namely, which of the drying factors affects liquid phase distribution during drying of porous media? Therefore, in these works, the pore network models are proposed, which are applicable for the slow isothermal drying of porous media.

In the works [24,25], associated heat and mass flows in the voids of complex geometry are considered. The conventional drying models, presented in the above works, are based on the assumption that a porous medium is a fictitious continuum, for which heat and mass balances are derived either by homogenization or by volume averaging. The pore network models are mainly developed because it is impossible to study transport phenomena at the pore level. Therefore, the exact description of a transfer in a porous medium is greatly simplified to the description of individual phases, i.e., gas and liquid.

In the works [25,26,27,30,31,32,33], the unsaturated moisture transfer processes in hygroscopic capillary-porous materials are simulated, demonstrating a wide pore size distribution. The pores are seen as computational nodes, where certain variables are computed, namely fluid pressure or vapor partial pressure. Transfer phenomena are described by one-dimensional approximations at the discrete pore level. Based on the mass balance at each node, two linear systems are formed to be solved numerically, in order to obtain partial vapor pressure in each gas pore (and in the boundary layer) and fluid pressure in each pore.

Correct determination of macroscopic parameters becomes the main problem to be solved. Through continuous advances in the imaging technology [34], as well as the use of methods of pore networks construction based on digital images of microstructures [35], it will only be a matter of time before these parameters are precisely determined based on the high performance pore network computations.

## 2. Materials and Methods

In this paper, a pore network model is used to study heat and mass transfer through a capillary-porous building material. In order to study temperature and moisture conditions of the capillary-porous material, a corresponding computational grid is formed, which is a system of rectangular channels, arranged in parallel to coordinate axes, and intersecting with each other.

Equivalent diameters of these channels correspond to the average pore diameter of the analyzed porous medium; a ratio of the total pore volume to the porous material volume corresponds to this material porosity.

### 2.1. Dispatch Model and Data

#### 2.1.1. Computational Grid

**Figure 3:**
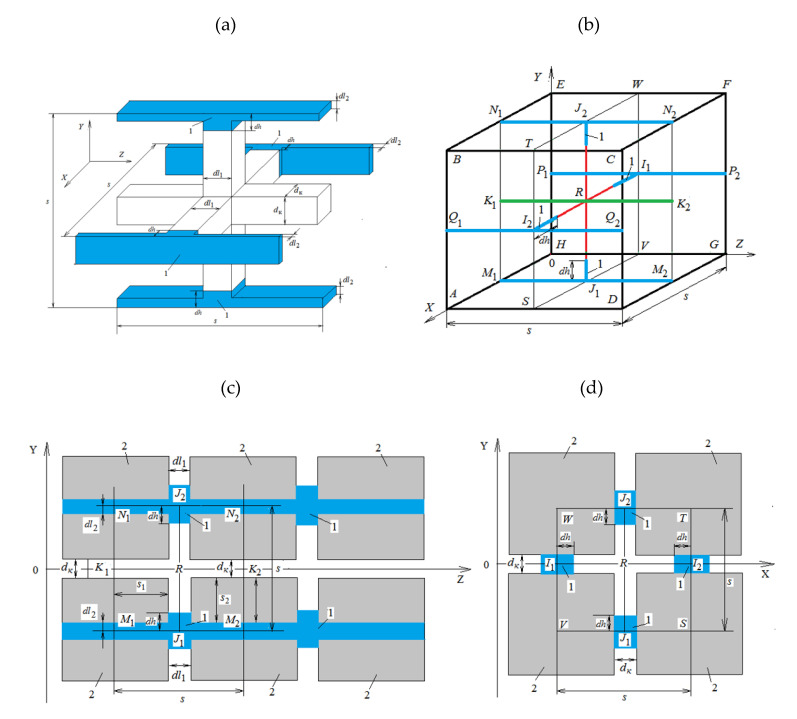
Cubic element of the computational scheme of the capillary-porous material network model: (**a**) volumetric image of a single computational cubic cell: (**b**) diagram of the lines of movement of material flows: (**c**) intersection of a cubic element by *Y0Z* plane: (**d**) intersection of a cubic element by *Y0X* plane.

One of the options to construct such a network is shown in Figure 3a–d.

The design model is based on a cubic element with *s* side. The pores are represented as intersecting square section channels. The side of the *d_k_* square corresponds to the known equivalent pore diameter of the material. The side length *s* of a cubic element is calculated from the condition

(1) $$s^{3}\varepsilon =3sd_{k}{}^{2}-2d_{k}{}^{3}$$

where *ε* is the known material porosity, expressing a ratio of pore volume to the total volume of the porous material.

The analyzed network model of heat and moisture transfer through a porous medium assumes that the most intensive vapor-air mixture (gas phase) and heat transfer occurs in the direction of *0Z* axis through *K*_1_*K*_2_ channels (Figure 3b,c), arranged parallel to this axis. These channels in sections are squares with *d_k_* side. Regarding heat and mass transfer through the building wall constructions, these channels are considered perpendicular to outer and inner surfaces of the building enclosure and connected to the inner and outer air media. According to this model, a liquid phase is arranged in the form of separate inclusions (mark 1 in Figure 3) in the network channels with *I*_1_*I*_2_*; J*_1_*J*_2_ axes are parallel to *0X* and *0Y* axes and perpendicular to *K*_1_*K*_2_ channels through which the gas phase is transferred. These inclusions of the liquid phase are in the form of rectangular columns. It is assumed that cross-sections of the channels, where liquid columns are arranged, are rectangles with *d_k_* and *dl*_1_ sides. It is assumed that columns with the liquid phase are interconnected by channels with *M*_1_*M*_2_, *N*_1_*N*_2_, *P*_1_*P*_2_ and *Q*_1_*Q*_2_ axes; they are parallel to the *0Z* axis. The channels, connecting liquid columns, also contain a liquid phase. According to the assumed model, liquid evaporates or condenses on the column surfaces, occupying sections *I*_1_*I*_2_ and *J*_1_*J*_2_ channels (Figure 3).

As a result of evaporation or condensation, the liquid mass in these columns, as well as their height, can vary with time. The liquid mass in the column-connecting channels is considered constant with time. Width of specified channels with *M*_1_*M*_2_, *N*_1_*N*_2_, *P*_1_*P*_2_ and *Q*_1_*Q*_2_ axes corresponds to *d_k_* value, and their height *dl*_2_ is calculated from the minimum possible moisture content of the liquid phase *w_l,min_* in the material, corresponding to conditions of the analyzed problem, *w_l,min_* value is determined by the minimum relative air humidity *φ_min_* in a porous material or in the external medium during the entire process of heat and mass transfer. This value is taken from the problem’s initial or boundary conditions. In order to determine *w_l,min_* from *φ_min_*, the sorption-desorption curve for an analyzed material should be used.

The liquid phase moisture content is considered as a ratio of the liquid mass in a certain volume of the porous material to this volume value. In the scope of considered cubic element, the moisture content is described by *w_l min_ = m_l_/s*^3^ expression, where ml is the liquid moisture mass, contained in this element. The liquid mass, contained in the considered channels with *M*_1_*M*_2_, *N*_1_*N*_2_, *P*_1_*P*_2_ and *Q*_1_*Q*_2_ axes, can be calculated as

$$m_{l,\min}=4sd_{K}dl_{2}\rho_{l}.$$

This value can also be obtained from the following expression

$$m_{l,\min}=w_{l,\min}\left(\phi_{\min}\right)s^{3}.$$

By making the last two expressions equal, we can get the width of channels *dl*_2_

$$dl_{2}=\frac{w_{l,\min}\left(\phi_{\min}\right)s^{2}}{4d_{K}\rho_{l}}\;.$$

With this configuration of the computational domain, the total pore volume in the considered cubic element is

$$V_{p}=d_{K}{}^{2}s+4dl_{1}d_{K}\times \left(\frac{s}{2}-\frac{d_{K}}{2}\right)+4\left(s-dl_{1}\right)d_{K}dl_{2}.$$

This value shall correspond to the specified material porosity *ε*. It follows from the condition (1) that

$$V_{p}=s^{3}\varepsilon =3sd_{k}{}^{2}-2d_{k}{}^{3}.$$

By making the last two expressions for *V_p_* equal, we can get the width of channels *dl*_1_, where liquid columns are arranged

$$dl_{1}=\frac{d_{K}\times \left(\frac{s}{2}-\frac{d_{K}}{2}\right)-dl_{2}\times s}{\left(\frac{s}{2}-\frac{d_{K}}{2}-dl_{2}\right)}.$$

This network model assumes that the heat and mass transfer processes proceed symmetrically relative to *ABCD*, *HEFG*, *BEFC* and *AHGD* planes. That is, there is no mass and heat transfer through these planes.

Intersections of these symmetry planes with the section, shown in Figure 3c, are represented by *N*_1_*N*_2_ and *M*_1_*M*_2_ segments, whereas intersections of symmetry planes with the section, shown in Figure 3d, correspond to *WT*, *TS*, *SV* and *VW* segments. The pore volume in a cubic element occupied by the liquid phase is

(2) $$V_{l}=4d_{k}\times dl_{2}\times s+4d_{k}\times dl_{1}\times \left(dh-dl_{2}\right).$$

If the moisture content of the liquid phase in a porous material is equal to *w_l_*, then its mass in the considered cubic element is

$$\delta m_{l}=V_{l}\times \rho_{l}=w_{l,0}\times s^{3}.$$

This equality, taking expression (2) into account, makes it possible to establish a relationship between the height of liquid columns *dh* and the material moisture content *w_l_*

(3) $$dh=\frac{w_{l}s^{3}+4\rho_{l}d_{k}dl_{2}\times \left(dl_{1}-s\right)}{4\rho_{l}d_{k}dl_{1}}.$$

#### 2.1.2. Transfer Model

In order to study the behavior of moisture content and temperature of the porous material with time, the mass and heat balance equations are formulated for the considered cubic elements, arranged sequentially in the direction of a *0Z* axis. The balance equations are formulated for discrete instants of time *τ_k_* with Δ*τ* interval.

The mass transfer in a gas phase, i.e., in a mixture of dry air and water vapor, occurs mainly in a channel with *K*_1_*K*_2_ axes by molecular diffusion and filtration. Mass transfer by the diffusion occurs due to mass concentration gradients (partial density) of dry air and vapor in a gas mixture, and it is described by Fick’s law:

(4) $$j_{a,dif}=-D_{va}\frac{\partial \rho_{a}}{\partial z}$$

(5) $$j_{v,dif}=-D_{va}\frac{\partial \rho_{a}}{\partial z}$$

where ρ*_v_*, [kg/m^3^] is partial density of water vapor in a mixture; ρ*_a_*, [kg/m^3^] is partial density of dry air in a mixture; *j_v_,_dif_*, [kg/(m^2^s)] is vapor flow density due to diffusion; *j_a_,_dif_*, [kg/(m^2^s)] is dry air flow density due to diffusion; *D_va_*, [m^2^/s] is the diffusion coefficient of water vapor and dry air in a gas mixture.

Density values of dry air and water vapor are calculated according to the ideal gas state equations;

(6) $$\rho_{a}=\frac{p_{a}}{R_{a}T_{g}}$$

(7) $$\rho_{v}=\frac{p_{v}}{R_{a}T_{g}}$$

where *p_a_*,*p_v_*, [Pa] is partial pressure of dry air and water vapor in a mixture; *R_a_*, *R_v_*, [J/(kg∙K)] are gas constants of dry air and water vapor; *T_g_*, [K] is gas mixture temperature. Besides, the gas medium (vapor-air mixture) transfer also occurs due to filtration.

Density values of vapor and air flows due to filtration are described by the Darcy equations;

(8) $$j_{a,fil}=-\rho_{a}\frac{K_{g}}{\mu_{g}}\frac{\partial p_{g}}{\partial z}$$

(9) $$j_{v,fil}=-\rho_{v}\frac{K_{g}}{\mu_{g}}\frac{\partial p_{g}}{\partial z}\;$$

where *K_g_*, [m^2^] is permeability coefficient of the porous material for a gas medium; *µ_g_*, [Pa∙s] is dynamic viscosity coefficient of a gas medium; $p_{g}=p_{a}+p_{v}$, [*Pa*] is vapor-air medium pressure.

The gas phase in a cubic element occupies the space of a channel with *K*_1_*K*_2_ axis, as well as part of the channel volumes with *I*_1_*I*_2_ and *J*_1_*J*_2_ axes (Figure 3). The volume, occupied by a gas phase, is calculated from the following expression:

(10) $$V_{g}{}_{i}^{k}=d_{K}{}^{2}s+4\left(\frac{s}{2}-\frac{d_{K}}{2}-dh_{i}^{k}\right)d_{K}dl_{1}$$

where $dh_{i}^{k}$ is liquid column height in the *i*-th element at *τ_k_* instant of time.

The balance equation of dry air mass in a cubic element with i number for τ_k_ instant of time is derived from the condition that air enters the considered element with diffusion $J^{+}{}_{a,dif}$ and filtration $J^{+}{}_{a,fil}$ flows from an adjacent element with *i* − 1 number through a surface with *d*_κ_^2^ area, and it is transferred to the next adjacent element with *i* + 1 number flows $J^{-}{}_{a,dif}$ and $J^{-}{}_{a,fil}$. In order to derive this equation, in expressions (4), (8) describing air flows by diffusion and filtration, the derivatives with respect to z variable are replaced by finite differences.

##### Dry Air Transport Dodel

Taking expression (6) into account, this equation is represented as:

(11) $$\frac{p_{a}{}_{,i}^{k}}{R_{a}T_{g}{}_{,i}^{k}}V_{g}{}_{i}^{k}-\frac{p_{a}{}_{,i}^{k-1}}{R_{a}T_{g}{}_{,i}^{k-1}}V_{g}{}_{i}^{k-1}=\left(J^{+}{}_{a,dif}-J^{-}{}_{a,dif}+J^{+}{}_{a,fil}-J^{-}{}_{a,fil}\right)d_{K}{}^{2}\Delta \tau$$

$$\begin{array}{c} J^{-}{}_{a,dif}=-\frac{D_{va,i+1/2}}{s}\left(\frac{p_{a}{}_{,i+1}^{k}}{R_{a}T_{g}{}_{,i+1}^{k}}-\frac{p_{a}{}_{,i}^{k}}{R_{a}T_{g}{}_{,i}^{k}}\right)\\ J^{+}{}_{a,fil}=-\frac{\rho_{a}{}_{,i-1/2}^{k}}{s}\frac{K_{g}}{\mu_{g}}\left(p_{a}{}_{,i}^{k}+p_{v}{}_{,i}^{k}-p_{a}{}_{,i-1}^{k}-p_{v}{}_{,i-1}^{k}\right)\\ J^{-}{}_{a,fil}=-\frac{\rho_{a}{}_{,i+1/2}^{k}}{s}\frac{K_{g}}{\mu_{g}}\left(p_{a}{}_{,i+1}^{k}+p_{v}{}_{,i+1}^{k}-p_{a}{}_{,i}^{k}-p_{v}{}_{,i}^{k}\right). \end{array}$$

This is the conservation equation for the local dry air mass. The left side of the equation is the mass difference in an elementary cubic cell between two successive points in time (through a time step), obtained from the law for an ideal gas. The right-hand side is recorded for the same times and consists of the difference in mass flows due to diffusion due to the concentration gradient and mass flow due to filtration due to the total pressure gradient. These two effects on the right side of expression (11) are not opposite to each other, but complement each other. Many researchers use this approach.

In this discrete equation, the values with the *i* index describe gas medium parameters in the considered an element of the porous material. Formally, it is considered that they refer to *R* node, located in the center of this element (Figure 3). Values with fractional indices are calculated as arithmetic (or weighted) mean values related to adjacent elements. Values with *k* index refer to the current moment of time, and those with the *k*−1 index to the previous one.

##### Water Vapor Transfer Model

The mass balance equation for water vapor is also based on the condition that vapor transfer through a cubic element occurs by diffusion and filtration in the direction of *0Z* axis. Diffusion and filtration water vapor flows are described by expressions (5) and (9), where the derivatives are replaced by finite differences.

Besides, it is considered that water vapor, evaporated from liquid column surfaces enters the gas medium with a diffusion flow $J^{+}{}_{l\_v,dif}$ through *I*_1_*I*_2_; *J*_1_*J*_2_ channels.

The vapor mass balance equation, considering expression (7), is written as:

(12) $$\begin{array}{l} \frac{p_{v}{}_{,i}^{k}}{R_{v}T_{g}{}_{,i}^{k}}V_{g}{}_{i}^{k}-\frac{p_{v}{}_{,i}^{k-1}}{R_{v}T_{g}{}_{,i}^{k-1}}V_{g}{}_{i}^{k-1}=\left(J^{+}{}_{v,dif}-J^{-}{}_{v,dif}+J^{+}{}_{v,fil}-J^{-}{}_{v,fil}\right)d_{K}{}^{2}\Delta \tau +\\ +4J_{l\_v,dif}dl_{1}d_{K}\Delta \tau , \end{array}$$

where

$$J^{+}{}_{v,dif}=-\frac{D_{va,i-1/2}}{s}\left(\frac{p_{v}{}_{,i}^{k}}{R_{v}T_{g}{}_{,i}^{k}}-\frac{p_{v}{}_{,i-1}^{k}}{R_{v}T_{g}{}_{,i-1}^{k}}\right);$$

$$J^{-}{}_{v,dif}=-\frac{D_{va,i+1/2}}{s}\left(\frac{p_{v}{}_{,i+1}^{k}}{R_{v}T_{g}{}_{,i+1}^{k}}-\frac{p_{v}{}_{,i}^{k}}{R_{v}T_{g}{}_{,i}^{k}}\right);$$

$$J^{+}{}_{v,fil}=-\frac{\rho_{v}{}_{,i-1/2}^{k}}{s}\frac{K_{g}}{\mu_{g}}\left(p_{a}{}_{,i}^{k}+p_{v}{}_{,i}^{k}-p_{a}{}_{,i-1}^{k}-p_{v}{}_{,i-1}^{k}\right);$$

$$J^{-}{}_{v,fil}=-\frac{\rho_{v}{}_{,i+1/2}^{k}}{s}\frac{K_{g}}{\mu_{g}}\left(p_{a}{}_{,i+1}^{k}+p_{v}{}_{,i+1}^{k}-p_{a}{}_{,i}^{k}-p_{v}{}_{,i}^{k}\right);$$

$$J_{l\_v,dif}=-\frac{D_{va,i}}{\frac{s}{2}-dh_{i}^{k}}\left(\frac{p_{v}{}_{,i}^{k}}{R_{v}T_{g}{}_{,i}^{k}}-\frac{p_{v\_l}{}_{,i}^{k}}{R_{v}T_{g\_l}{}_{,i}^{k}}\right)+\frac{p_{v\_l}{}_{,i}^{k}}{R_{v}T_{g\_l}{}_{,i}^{k}}u_{S};$$

$\left(\frac{s}{2}-dh_{i}^{k}\right)$ is a distance from the surface of liquid columns to *R* point; *u_s_* is Stefan’s speed; $p_{v\_l}{}_{,i}^{k}$ is the partial pressure of water vapor directly above the surface of liquid columns; $T_{g\_l}{}_{,i}^{k}$ is the temperature of the liquid column surface, where liquid is evaporated from.

##### Liquid (Water) Transfer Model

Liquid phase transfer in the channels with *M*_1_*M*_2_, *N*_1_*N*_2_, *P*_1_*P*_2_ and *Q*_1_*Q*_2_ axes occurs due to filtration, resulting from the action of pressure gradient in a liquid medium. This filtration flow is described by the Darcy equation

(13) $$j_{l}=-\rho_{l}\frac{K_{l}}{\mu_{l}}\frac{\partial p_{l}}{\partial z}$$

where *j_l_* [kg/(m^2^s)] is density of the filtration fluid flow; *p_l_* [Pa] is pressure in a liquid phase; *µ_l_* [Pa∙s] is the dynamic coefficient of medium liquid viscosity; [Pa∙s] is the dynamic coefficient of medium liquid viscosity; *ρ_l_* [kg/m^3^] is liquid density; *K_l_* [m^2^] is the permeability coefficient of the porous material for a liquid medium. Pressure in a liquid phase is defined as the difference between a vapor-gas medium and capillary pressure:

$$p_{l}=p_{g}-p_{c}.$$

Considering this expression, Equation (13) can be written as

(14) $$j_{l}=\rho_{l}\frac{K_{l}}{\mu_{l}}\frac{\partial p_{c}}{\partial z}$$

since it can be assumed that $\frac{\partial p_{g}}{\partial z}<<\frac{\partial p_{c}}{\partial z}$.

Capillary pressure *p_c_* depends on the specific moisture content *w_l_*. In this regard, derivative $\frac{\partial p_{c}}{\partial z}$ in the expression (16) is replaced by $\frac{\partial p_{c}}{\partial z}=\frac{dp_{c}}{dw_{l}}\frac{dw_{l}}{dh}\frac{\partial h}{\partial z}$. Derivative $\frac{dp_{c}}{dw_{l}}$ is determined from the experimental dependence of capillary pressure *p_c_* on the specific moisture content *w_l_*, derivative $\frac{dw_{l}}{dh}$ is calculated from the expression (3):

$$\frac{dw_{l}}{dh}=\frac{4dl_{1}d_{K}\rho_{l}}{s^{3}}=C_{h}$$

Accordingly, the mass balance equation for a liquid phase is derived

(15) $$\rho_{l}\left(dh_{i}^{k}-dh_{i}^{k-1}\right)dl_{1}d_{K}=\left(J^{+}{}_{l,fil}-J^{-}{}_{l,fil}\right)dl_{2}d_{K}\Delta \tau -J_{l\_v,dif}dl_{1}d_{K}\Delta \tau ;$$

where

$$J^{+}{}_{l,fil}=\frac{\rho_{l}}{\mu_{l}}{C_{h}\left(K_{l}\frac{dp_{c}}{dw_{l}}\right)|}_{i-1/2}\frac{h_{i}^{k}-h_{i-1}^{k}}{s};$$

$$J^{-}{}_{l,fil}=\frac{\rho_{l}}{\mu_{l}}{C_{h}\left(K_{l}\frac{dp_{c}}{dw_{l}}\right)|}_{i+1/2}\frac{h_{i+1}^{k}-h_{i}^{k}}{s}.$$

Equation (17), as well as Equation (13) for water vapor consider the diffusion transfer $J_{l\_v,dif}$ of evaporated moisture from liquid column surfaces into a gas phase.

The partial pressure of water vapor above the surface of liquid columns is calculated as

$$p_{v\_l}{}_{,i}^{k}=p_{sut}\left(T_{g\_l}{}_{,i}^{k}\right)\cdot \varphi \left(w_{l}{}_{,i}^{k},T_{g\_l}{}_{,i}^{k}\right),$$

where $p_{sut}\left(T_{g\_l}{}_{,i}^{k}\right)$ is saturation pressure, corresponding to the surface temperature of liquid columns; $\varphi \left(w_{l}{}_{,i}^{k},T_{g\_l}{}_{,i}^{k}\right)$ is relative air humidity, corresponding to specific moisture content $w_{l}{}_{,i}^{k}$. This dependence is determined from the sorption-desorption isotherm for a specified material.

##### Model of Heat Transfer in a Vapor-Air Medium

The energy conservation equation for volume $V_{g}{}_{i}^{k}$ of the vapor-air mixture is based on the condition that heat enters this volume by convection *Q_g_conv_* and heat conductivity *Q_g_cond_*. The heat convective flows *Q_g_conv_* are created by diffusion and filtration flows of dry air and water vapor.

**Figure 4:**
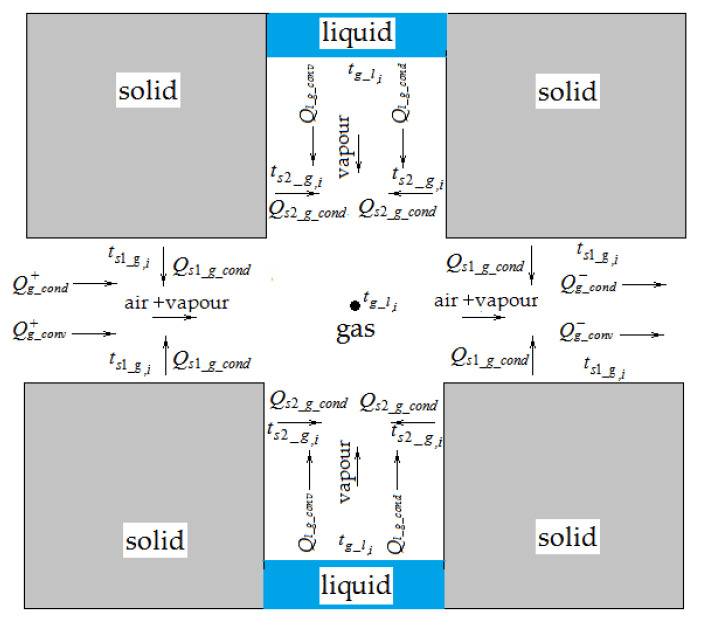
Scheme of heat transfer for the gas phase in an elementary cubic element.

In addition, the heat *Q_l_g_conv_* is transferred by convection into a gas medium with moisture flow, evaporated from liquid column surfaces. The heat flow with heat conductivity that *Q_g_cond_* generates is due to the presence of a temperature gradient in a gas medium along the *0Z* axis. By means of heat conductivity, the heat *Q_l_g_cond_* also enters the considered volume from the surface of liquid columns, resulting from temperature differences between a gas medium and a liquid phase. Besides, the heat *Q_s_g_cond_* = *Q_s_*_1*_g_cond*_ + *Q_s_*_2*_g_cond*_ centers a gas medium from the surfaces of pore walls by means of heat conductivity. A block diagram of the movement of heat and material flows (and their corresponding designations) for the central nodal part of a single elementary cubic element of material, which is shown in Figure 3c, which in an enlarged form is shown in Figure 4.

Considering the above, the heat balance equation for a gas medium is derived:

$$\begin{array}{l} V_{g}{}_{i}^{k}\left(I_{a}{}_{i}^{k}+I_{v}{}_{i}^{k}\right)=V_{g}{}_{i}^{k-1}\left(I_{a}{}_{i}^{k-1}+I_{v}{}_{i}^{k-1}\right)+Q^{+}{}_{g\_conv}-Q^{-}{}_{g\_conv}+Q^{+}{}_{g\_cond}-Q^{-}{}_{g\_cond}+Q_{l\_g\_conv}+Q_{l\_g\_cond}+\\ +Q_{s1\_g\_cond}+Q_{s2\_g\_cond}; \end{array}$$

where

$Q^{+}{}_{g\_conv}=\left(J^{+}{}_{a,dif}+J^{+}{}_{a,fil}\right)I_{a}{,}_{i-1/2}^{k}d_{K}{}^{2}\Delta \tau +\left(J^{+}{}_{v,dif}+J^{+}{}_{v,fil}\right)I_{v}{,}_{i-1/2}^{k}d_{K}{}^{2}\Delta \tau ;$
$Q^{-}{}_{g\_conv}=\left(J^{-}{}_{a,dif}+J^{-}{}_{a,fil}\right)I_{a}{,}_{i+1/2}^{k}d_{K}{}^{2}\Delta \tau +\left(J^{-}{}_{v,dif}+J^{-}{}_{v,fil}\right)I_{v}{,}_{i+1/2}^{k}d_{K}{}^{2}\Delta \tau ;$
$Q^{+}{}_{g\_cond}=q{+}_{g}d_{K}{}^{2}\Delta \tau ;$$Q^{-}{}_{g\_cond}=q^{-}{}_{g}d_{K}{}^{2}\Delta \tau ;$
$Q_{l\_g\_conv}=4J_{l\_v,dif}I_{l\_v}{,}_{i}^{k}dl_{1}d_{K}\Delta \tau ;$$Q_{l\_g\_cond}=4q^{+}{}_{l\_g}dl_{1}d_{K}\Delta \tau ;$
$Q_{s1\_g\_cond}=4q^{+}{}_{s1\_g}\left(s-dl_{1}\right)d_{K}\Delta \tau ;$$Q_{s2\_g\_cond}=4q^{+}{}_{s2\_g}f_{s2\_g}\Delta \tau +4q^{+}{}_{s3\_g}f_{s3\_g}\Delta \tau$.

After substituting these expressions into the heat balance equation for gas, we get:

(16) $$\begin{array}{l} V_{g}{}_{i}^{k}\left(I_{a}{}_{i}^{k}+I_{v}{}_{i}^{k}\right)=V_{g}{}_{i}^{k-1}\left(I_{a}{}_{i}^{k-1}+I_{v}{}_{i}^{k-1}\right)+\\ +\left(J^{+}{}_{a,dif}+J^{+}{}_{a,fil}\right)I_{a}{,}_{i-1/2}^{k}d_{K}{}^{2}\Delta \tau -\left(J^{-}{}_{a,dif}+J^{-}{}_{a,fil}\right)I_{a}{,}_{i+1/2}^{k}d_{K}{}^{2}\Delta \tau +\\ +\left(J^{+}{}_{v,dif}+J^{+}{}_{v,fil}\right)I_{v}{,}_{i-1/2}^{k}d_{K}{}^{2}\Delta \tau -\left(J^{-}{}_{v,dif}+J^{-}{}_{v,fil}\right)I_{v}{,}_{i+1/2}^{k}d_{K}{}^{2}\Delta \tau +\\ +4J_{l\_v,dif}I_{l\_v}{,}_{i}^{k}dl_{1}d_{K}\Delta \tau +q^{+}{}_{g}d_{K}{}^{2}\Delta \tau -q^{-}{}_{g}d_{K}{}^{2}\Delta \tau \\ +4q^{+}{}_{l\_g}dl_{1}d_{K}\Delta \tau +4q^{+}{}_{s1\_g}\left(s-dl_{1}\right)d_{K}\Delta \tau +4q^{+}{}_{s2\_g}f_{s2\_g}\Delta \tau +4q^{+}{}_{s3\_g}f_{s3\_g}\Delta \tau \end{array}$$

where

$I_{a}{}_{i}^{k}=C_{a}t_{g}{}_{i}^{k}\frac{p_{a}{}_{i}^{k}}{R_{a}T_{g}{}_{i}^{k}}$; $I_{v}{}_{i}^{k}=\left[C_{w}t_{н}\left(p_{v}{}_{i}^{k}\right)+r_{v}+C_{v}\left(t_{g}{}_{i}^{k}-t_{н}\left(p_{v}{}_{i}^{k}\right)\right)\right]\frac{p_{v}{}_{i}^{k}}{R_{v}T_{g}{}_{i}^{k}}$;
$I_{l\_v}{,}_{i}^{k}=C_{w}t_{g\_l}{}_{,i}^{k}+r_{v}$; $q^{+}{}_{g}=-\lambda_{g}\frac{t_{g}{}_{i}^{k}-t_{g}{}_{i-1}^{k}}{s}$; $q^{-}{}_{g}=-\lambda_{g}\frac{t_{g}{}_{i+1}^{k}-t_{g}{}_{i}^{k}}{dz}$;
$q^{+}{}_{l\_g}=-\lambda_{g}\frac{t_{g}{}_{,i}^{k}-t_{g\_l}{}_{,i}^{k}}{\left(\frac{s}{2}-dh_{i}^{k}\right)}$; $q^{+}{}_{s1\_g}=-\lambda_{g}\frac{t_{g}{}_{,i}^{k}-t_{s1\_g}{}_{,i}^{k}}{d_{K}/2}$; $q^{+}{}_{s2\_g}=-\lambda_{g}\frac{t_{g}{}_{,i}^{k}-t_{s2\_g}{}_{,i}^{k}}{dl_{1}/2}$;
$q^{+}{}_{s3\_g}=-\lambda_{g}\frac{t_{g}{}_{,i}^{k}-t_{s2\_g}{}_{,i}^{k}}{d_{K}/2}$; $f_{s2\_g}=2d_{K}\left(\frac{s}{2}-\frac{d_{K}}{2}-dh_{i}^{k}\right)$; $f_{s3\_g}=2dl_{1}\left(\frac{s}{2}-\frac{d_{K}}{2}-dh_{i}^{k}\right)$;

$t_{s1\_g}{}_{,i}^{k}$ is temperature [°C] of the channel wall surfaces with *K*_1_*K*_2_ axis, which is in contact with a vapor-gas medium; $t_{s2\_g}{}_{,i}^{k}$ is the temperature of channel wall surfaces with *I*_1_*I*_2_ and *J*_1_*J*_2_ axes, which are in contact with a vapor-gas medium; $t_{н}\left(p_{v}{}_{i}^{k}\right)$ is saturation temperature, corresponding to vapor pressure; $p_{v}{}_{i}^{k}$; *C_a_*; *C_v_*; *C_w_*, [J/(kg∙K)] are specific heat capacity values of dry air, water vapor and water; *r_v_*, [J/kg] is specific heat of vapor formation; *λ_g_*, [W/(m∙K)] is the heat conductivity coefficient of a vapor-gas mixture; $f_{s2\_g}$; $f_{s3\_g}$ are contact surfaces of a vapor-gas mixture with channel walls with *I*_1_*I*_2_; *J*_1_*J*_2_ axes *I*_1_*I*_2_; *J*_1_*J*_2_.

##### Model of Heat Transfer in the Liquid Phase

The energy conservation equations for a liquid phase are derived for liquid volume $V_{l}=d_{K}dl_{2}s+\left(dh_{i}^{k}-dl_{2}\right)d_{K}dl_{1}$, including channel volumes, *dl*_2_, high, containing a constant liquid volume, and volumes of liquid columns, $dh_{i}^{k}-dl_{2}$ varying with time. If the liquid temperature value in *J*_1_; *J*_2_; *I*_1_; *I*_2_ nodes (Figure 1) in the design element with i number is $t_{w}{}_{,i}^{k}$, then the heat content in this liquid volume at the time step *k* is calculated from the expression $Q_{l}{}_{,i}^{k}=C_{w}\rho_{w}t_{w}{}_{,i}^{k}\left[d_{K}dl_{2}s+\left(dh_{i}^{k}-dl_{2}\right)d_{K}dl_{1}\right]$.

Through channels with *M*_1_*M*_2_, *N*_1_*N*_2_, *P*_1_*P*_2_ *Q*_1_*Q*_2_ axes, where the liquid fraction is located, the heat transfer is performed by means of heat conductivity due to the temperature gradient, as well as by convection with filtration liquid flows.

From the surfaces of liquid columns, heat is removed from the considered volume by means of heat conductivity and convection with liquid flow, evaporating from the column surface and transferred into a vapor-air mixture. Heat is also transferred from liquid to channel walls by *M*_1_*M*_2_, *N*_1_*N*_2_, *P*_1_*P*_2_ and *Q*_1_*Q*_2_ axes, as well as to channel walls with *J*_1_*J*_2_ and *I*_1_*I*_2_ axes, where the liquid columns are located. Thus, the energy conservation equation for the liquid fraction is represented as:

(17) $$\begin{array}{l} C_{w}\rho_{w}t_{w}{}_{,i}^{k}V_{l}{}_{,i}^{k}=C_{w}\rho_{w}t_{w}{}_{,i}^{k-1}V_{l}{}_{,i}^{k-1}-q^{-}{}_{l\_g}dl_{1}d_{K}\Delta \tau -J_{l\_v,dif}C_{w}t_{g\_l}{}_{,i}^{k}dl_{1}d_{K}\Delta \tau \\ +\left(q^{+}{}_{w}-q^{-}{}_{w}\right)d_{K}dl_{2}\Delta \tau +\left(J^{+}{}_{l,fil}C_{w}t_{w}{}_{i-1/2}^{k}-J^{-}{}_{l,fil}C_{w}t_{w}{}_{i+1/2}^{k}\right)dl_{2}d_{K}\Delta \tau -\\ -q^{-}{}_{l\_s1}f_{s1\_l}\Delta \tau +q^{-}{}_{l\_s2}f_{s2\_l}\Delta \tau +q^{-}{}_{l\_s3}f_{s3\_l}\Delta \tau \end{array}$$

where

$q^{-}{}_{l\_g}=-\lambda_{w}\frac{t_{g\_l}{}_{,i}^{k}-t_{w}{}_{,i}^{k}}{dh_{i}^{k}}$; $q^{+}{}_{w}=-\lambda_{w}\frac{t_{w}{}_{i}^{k}-t_{w}{}_{i-1}^{k}}{s}$; $q^{-}{}_{w}=-\lambda_{w}\frac{t_{w}{}_{i+1}^{k}-t_{w}{}_{i}^{k}}{s}$;
$q^{-}{}_{l\_s1}=-\lambda_{w}\frac{t_{w-s1}{}_{,i}^{k}-t_{w}{}_{,i}^{k}}{dl_{2}}$; $q^{-}{}_{l\_s2}=-\lambda_{w}\frac{t_{w}{}_{,i}^{k}-t_{w-s2}{}_{,i}^{k}}{dl_{1}/2}$; $q^{-}{}_{l\_s3}=-\lambda_{w}\frac{t_{w}{}_{,i}^{k}-t_{w-s2}{}_{,i}^{k}}{d_{K}/2}$;
$f_{s1\_l}=\left(2dl_{2}+d_{K}\right)\left(s-dl_{1}\right)$; $f_{s2\_l}=2d_{K}\left(dh_{i}^{k}-dl_{2}\right)$; $f_{s3\_l}=2dl_{1}\left(dh_{i}^{k}-dl_{2}\right)$.

$t_{w-s1}{}_{,i}^{k}$ is the temperature ([°C]) of channel wall surfaces with *M*_1_*M*_2_; *N*_1_*N*_2_; *P*_1_*P*_2_; *Q*_1_*Q*_2_ axes, which are in contact with a liquid phase; $t_{w-s2}{}_{,i}^{k}$ is temperature of channel wall surfaces with *I*_1_*I*_2_; *J*_1_*J*_2_ axes, which are in contact with a liquid medium; *λ_w_*, W/(m K) is liquid heat conductivity coefficient; $f_{s1\_l}$ is a contact surface of a liquid phase with channel walls, through which the liquid is filtered $f_{s2\_l}$; $f_{s3\_l}$- are contact surfaces of a vapor-gas mixture with channel walls, where the liquid column is located.

##### Heat Transfer Model in a Solid Structure

The energy conservation equation for a solid fraction of the considered element, occupying volume $V_{s}=s^{3}\left(1-\varepsilon \right)$, is derived taking into account the fact that heat transfer occurs along a solid body in the direction of *0Z* axis by means of heat conductivity.

The heat flow by means of heat conductivity enters the cubic element and leaves it through the face with the area of $f_{s}=s^{2}-d_{K}{}^{2}-4d_{K}dl_{2}$. On the surfaces of channel walls with the *K*_1_*K*_2_ axis, the heat exchange of a solid fraction with a gas medium occurs. On channel walls with *I*_1_*I*_2_ and *J*_1_*J*_2_ axes, the heat exchange occurs with a gas phase, present in these channels, as well as with liquid columns. On channel walls with *M*_1_*M*_2_, *N*_1_*N*_2_, *P*_1_*P*_2_ and *Q*_1_*Q*_2_ axes, there is a heat exchange of solid phase with a liquid medium.

Thus, the energy conservation equation for the solid fraction is represented as:

(18) $$\begin{array}{l} C_{s}\rho_{s}t_{s}{}_{,i}^{k}V_{s}=C_{s}\rho_{s}t_{s}{}_{,i}^{k-1}V_{s}+q^{+}{}_{s}f_{s}\Delta \tau -q^{-}{}_{s}f_{s}\Delta \tau -4q^{-}{}_{s1\_g}\left(s-dl_{1}\right)d_{K}\Delta \tau +\\ +4q^{+}{}_{s1\_l}f_{s1\_l}\Delta \tau -4q^{+}{}_{l\_s2}f_{s2\_l}\Delta \tau -4q^{+}{}_{l\_s3}f_{s3\_l}\Delta \tau -\\ -4q^{-}{}_{s2\_g}f_{s2\_g}\Delta \tau -4q^{-}{}_{s3\_g}f_{s3\_g}\Delta \tau \end{array}$$

where

$q^{+}{}_{s}=-\lambda_{s}\frac{t_{s}{}_{i}^{k}-t_{s}{}_{i-1}^{k}}{s}$; $q^{-}{}_{s}=-\lambda_{s}\frac{t_{s}{}_{i+1}^{k}-t_{s}{}_{i}^{k}}{s}$; $q^{-}{}_{s1\_g}=-\lambda_{s}\frac{t_{s1\_g}{}_{,i}^{k}-t_{s}{}_{,i}^{k}}{s_{2}/2}$; $q^{+}{}_{s1\_l}=-\lambda_{s}\frac{t_{s}{}_{,i}^{k}-t_{w-s1}{}_{,i}^{k}}{s_{2}/2}$;
$s_{2}=0,5\left(s-d_{K}\right)-dl_{2}$;
$q^{+}{}_{l\_s2}=-\lambda_{s}\frac{t_{w-s2}{}_{,i}^{k}-t_{s}{}_{,i}^{k}}{s_{1,1}/2}$; $q^{+}{}_{l\_s3}=-\lambda_{s}\frac{t_{w-s2}{}_{,i}^{k}-t_{s}{}_{,i}^{k}}{s_{1,2}/2}$;
$q^{-}{}_{s2\_g}=-\lambda_{s}\frac{t_{s2\_g}{}_{,i}^{k}-t_{s}{}_{,i}^{k}}{s_{1,1}/2}$; $q^{-}{}_{s3\_g}=-\lambda_{s}\frac{t_{s2\_g}{}_{,i}^{k}-t_{s}{}_{,i}^{k}}{s_{1,2}/2}$;
$s_{1,1}=0,5\left(s-dl_{1}\right)$; $s_{1,2}=0,5\left(s-d_{K}\right)$.

The temperature-moisture state of a capillary-porous material is described by a system of equations for the mass and energy conservation: (11); (12); (15); (16); (17); (18). This system of equations is written for all cubic elements with the numbers *I =* 1*...N*.

Its solution makes it possible to calculate the values of network functions describing: partial pressure of dry air $p_{a}{}_{,i}^{k}$; partial pressure of water vapor $p_{v}{}_{,i}^{k}$; height of liquid columns $dh_{i}^{k}$; vapor-air mixture temperature $t_{g}{}_{i}^{k}$; liquid phase temperature $t_{w}{}_{,i}^{k}$ and solid fraction temperature of a porous material $t_{s}{}_{,i}^{k}$. Except for indicated values, this system of equations also contains: liquid column surface temperature $t_{g\_l}{}_{,i}^{k}$; surface temperature of channel walls with *K*_1_*K*_2_ axis, which is in contact with a vapor-gas medium $t_{s1\_g}{}_{,i}^{k}$; surface temperature of channel walls with *I*_1_*I*_2_; *J*_1_*J*_2_ axes, which are in contact with a vapor-gas medium $t_{s2\_g}{}_{,i}^{k}$; surface temperature of channel walls with *M*_1_*M*_2_; *N*_1_*N*_2_; *P*_1_*P*_2_; *Q*_1_*Q*_2_ axes, which are in contact with a liquid phase $t_{w-s1}{}_{,i}^{k}$ and surface temperature of channel walls with *I*_1_*I*_2_ and *J*_1_*J*_2_ axes, which are in contact with a liquid medium $t_{w-s2}{}_{,i}^{k}$. In order to determine specified temperature values on the medium contact surfaces, the matching conditions are used.

#### 2.1.3. The Matching Conditions on the Surfaces

The matching conditions on the surfaces of liquid columns, where a vapor-air mixture contacts with the liquid, and from which evaporation (condensation) occurs, are as follows:

$$q^{-}{}_{l\_g}=r_{v}J_{l\_v,dif}+q^{+}{}_{l\_g}$$

or

$$-\lambda_{w}\frac{t_{g\_l}{}_{,i}^{k}-t_{w}{}_{,i}^{k}}{dh_{i}^{k}}=r_{v}J_{l\_v,dif}-\lambda_{g}\frac{t_{g}{}_{,i}^{k}-t_{g\_l}{}_{,i}^{k}}{0,5s-dh_{i}^{k}}.$$

The value is determined from this expression: $t_{g\_l}{}_{,i}^{k}$:

$$t_{g\_l}{}_{,i}^{k}=\frac{\frac{\lambda_{w}}{dh_{i}^{k}}}{\left(\frac{\lambda_{g}}{0,5s-dh_{i}^{k}}+\frac{\lambda_{w}}{dh_{i}^{k}}\right)}t_{w}{}_{,i}^{k}+\frac{\frac{\lambda_{g}}{0,5s-dh_{i}^{k}}}{\left(\frac{\lambda_{g}}{0,5s-dh_{i}^{k}}+\frac{\lambda_{w}}{dh_{i}^{k}}\right)}t_{g}{}_{,i}^{k}-\frac{r_{v}J_{l\_v,dif}}{\left(\frac{\lambda_{g}}{0,5s-dh_{i}^{k}}+\frac{\lambda_{w}}{dh_{i}^{k}}\right)}.$$

The matching condition on channel walls with *M*_1_*M*_2_; *N*_1_*N*_2_; *P*_1_*P*_2_; *Q*_1_*Q*_2_ axes, which are in contact with a liquid phase, are represented as:

$$q^{-}{}_{l\_s1}=q^{+}{}_{s1\_l}$$

or

$$-\lambda_{w}\frac{t_{w-s1}{}_{,i}^{k}-t_{w}{}_{,i}^{k}}{dl_{2}}=-\lambda_{s}\frac{t_{s}{}_{,i}^{k}-t_{w-s1}{}_{,i}^{k}}{0,5s_{2}}.$$

This expression determines $t_{w-s1}{}_{,i}^{k}$

$$t_{w-s1}{}_{,i}^{k}=\frac{\frac{\lambda_{w}}{dl_{2}}}{\left(\frac{\lambda_{s}}{0,5s_{2}}+\frac{\lambda_{w}}{dl_{2}}\right)}t_{w}{}_{,i}^{k}+\frac{\frac{\lambda_{s}}{0,5s_{2}}}{\left(\frac{\lambda_{s}}{0,5s_{2}}+\frac{\lambda_{w}}{dl_{2}}\right)}t_{s}{}_{,i}^{k}.$$

The matching condition on channel walls with *K*_1_*K*_2_ axis, where the heat exchange of a vapor-gas mixture with a solid phase of the porous material occurs, is as follows:

$$q^{+}{}_{s1\_g}=q^{-}{}_{s1\_g}$$

or

$$-\lambda_{g}\frac{t_{g}{}_{,i}^{k}-t_{s1\_g}{}_{,i}^{k}}{0,5d_{K}}=-\lambda_{s}\frac{t_{s1\_g}{}_{,i}^{k}-t_{s}{}_{,i}^{k}}{0,5s_{2}}.$$

This equation determines the contact surface temperature of channel walls with *K*_1_*K*_2_ axis with a vapor-air medium

$$t_{s1\_g}{}_{,i}^{k}=\frac{\frac{\lambda_{s}}{s_{2}}}{\left(\frac{\lambda_{g}}{d_{K}}+\frac{\lambda_{s}}{s_{2}}\right)}t_{s}{}_{,i}^{k}+\frac{\frac{\lambda_{g}}{d_{K}}}{\left(\frac{\lambda_{g}}{d_{K}}+\frac{\lambda_{s}}{s_{2}}\right)}t_{g}{}_{,i}^{k}.$$

In order to determine the surface temperature $t_{s2\_g}{}_{,i}^{k}$ of channel walls with *I*_1_*I*_2_; *J*_1_*J*_2_ axes, which are in contact with a vapor-gas medium, the matching conditions are represented as

$$q^{+}{}_{s2\_g}f_{s2\_g}+q^{+}{}_{s3\_g}f_{s3\_g}=q^{-}{}_{s2\_g}f_{s2\_g}+q^{-}{}_{s3\_g}f_{s3\_g}$$

or with consideration of the above expressions

$$\begin{array}{l} -\lambda_{g}\frac{t_{g}{}_{,i}^{k}-t_{s2\_g}{}_{,i}^{k}}{dl_{1}/2}2d_{K}\left(\frac{s}{2}-\frac{d_{K}}{2}-dh_{i}^{k}\right)-\lambda_{g}\frac{t_{g}{}_{,i}^{k}-t_{s2\_g}{}_{,i}^{k}}{d_{K}/2}2dl_{1}\left(\frac{s}{2}-\frac{d_{K}}{2}-dh_{i}^{k}\right)=\\ =-\lambda_{s}\frac{t_{s2\_g}{}_{,i}^{k}-t_{s}{}_{,i}^{k}}{s_{1,1}/2}2d_{K}\left(\frac{s}{2}-\frac{d_{K}}{2}-dh_{i}^{k}\right)-\lambda_{s}\frac{t_{s2\_g}{}_{,i}^{k}-t_{s}{}_{,i}^{k}}{s_{1,2}/2}2dl_{1}\left(\frac{s}{2}-\frac{d_{K}}{2}-dh_{i}^{k}\right) \end{array}.$$

From the presented expression, it follows that

$$t_{s2\_g}{}_{,i}^{k}=\frac{\lambda_{s}\left(\frac{d_{K}}{s_{1,1}}+\frac{dl_{1}}{s_{1,2}}\right)}{\left(\lambda_{g}\left(\frac{d_{K}}{dl_{1}}+\frac{dl_{1}}{d_{K}}\right)+\lambda_{s}\left(\frac{d_{K}}{s_{1,1}}+\frac{dl_{1}}{s_{1,2}}\right)\right)}t_{s}{}_{,i}^{k}+\frac{\lambda_{g}\left(\frac{d_{K}}{dl_{1}}+\frac{dl_{1}}{d_{K}}\right)}{\left(\lambda_{g}\left(\frac{d_{K}}{dl_{1}}+\frac{dl_{1}}{d_{K}}\right)+\lambda_{s}\left(\frac{d_{K}}{s_{1,1}}+\frac{dl_{1}}{s_{1,2}}\right)\right)}t_{g}{}_{,i}^{k}.$$

The surface temperature $t_{w-s2}{}_{,i}^{k}$ of channel walls with *I*_1_*I*_2_; *J*_1_*J*_2_ axes, which are in contact with a liquid medium, is determined using the matching condition

$$q^{+}{}_{l\_s2}f_{s2\_l}+q^{+}{}_{l\_s3}f_{s3\_l}=q^{-}{}_{l\_s2}f_{s2\_l}+q^{-}{}_{l\_s3}f_{s3\_l}.$$

which, taking above expressions into account, is represented as

$$\begin{array}{l} -\lambda_{s}\frac{t_{w-s2}{}_{,i}^{k}-t_{s}{}_{,i}^{k}}{s_{1,1}/2}2d_{K}\left(dh_{i}^{k}-dl_{2}\right)-\lambda_{s}\frac{t_{w-s2}{}_{,i}^{k}-t_{s}{}_{,i}^{k}}{s_{1,2}/2}2dl_{1}\left(dh_{i}^{k}-dl_{2}\right)=\\ =-\lambda_{w}\frac{t_{w}{}_{,i}^{k}-t_{w-s2}{}_{,i}^{k}}{dl_{1}/2}2d_{K}\left(dh_{i}^{k}-dl_{2}\right)-\lambda_{w}\frac{t_{w}{}_{,i}^{k}-t_{w-s2}{}_{,i}^{k}}{d_{K}/2}2dl_{1}\left(dh_{i}^{k}-dl_{2}\right) \end{array}.$$

From this expression, it follows that

$$t_{w-s2}{}_{,i}^{k}=\frac{\lambda_{s}\left(\frac{d_{K}}{s_{1,1}}+\frac{dl_{1}}{s_{1,2}}\right)}{\left(\lambda_{w}\left(\frac{d_{K}}{dl_{1}}+\frac{dl_{1}}{d_{K}}\right)+\lambda_{s}\left(\frac{d_{K}}{s_{1,1}}+\frac{dl_{1}}{s_{1,2}}\right)\right)}t_{s}{}_{,i}^{k}+\frac{\lambda_{w}\left(\frac{d_{K}}{dl_{1}}+\frac{dl_{1}}{d_{K}}\right)}{\left(\lambda_{w}\left(\frac{d_{K}}{dl_{1}}+\frac{dl_{1}}{d_{K}}\right)+\lambda_{s}\left(\frac{d_{K}}{s_{1,1}}+\frac{dl_{1}}{s_{1,2}}\right)\right)}t_{w}{}_{,i}^{k}.$$

### 2.2. Condition for Solving Equations

In order to solve the problem of heat and mass transfer dynamics in the considered formulation, the initial and boundary conditions for presented equations should be formulated. The initial distribution of temperature *t_0_* and moisture content *w_l,_*_0_ over the material thickness can be set as initial conditions. The boundary conditions shall reflect ambient temperature *t_∞_* and a certain indicator of its moisture condition: relative air humidity *φ_∞_* or partial pressure of water vapor *p_v,∞_* or its concentration *ρ_v,∞_* in air. Also, the total pressure of a vapor-air mixture outside the material *p_g,_*_∞_ = *p_a,_*_∞_ + *p_v,_*_∞_, should be set; it usually corresponds to the atmospheric pressure.

## 3. Results

As an example, the change in temperature and moisture condition in time of a porous material, *Z* = 0.1 [m] thick was analyzed. Its porosity is ε = 0.157. Thermophysical properties of the considered material correspond to properties of a ceramic brick. Permeability coefficient for gaseous medium $K_{g}=2.2\times 10^{-13}$, [m^2^]. For the dependences of the permeability coefficients and capillary pressure for the liquid in the material on the moisture content, the data given in [9] were used. Note that the values of capillary pressure and the coefficient of permeability of a liquid in a material depend significantly on its moisture content. For the considered range of changes in moisture content *w* = 3...60 [kg/m^3^] capillary pressure, respectively, varied within *p_c_* = 9.5 × 10^6^...0.1 × 10^6^ [*Pa*], and the ratio of the permeability coefficient (for liquid) to its dynamic coefficient viscosity varied in the range $K_{l}/\mu_{l}=4.0\times 10^{-}{}^{16}\ldots 6.7\times 10^{-11}$ [m^2^] [9]. The heat capacity and thermal conductivity coefficients for each phase were chosen to be constant and, accordingly, equal: *C_a_* = 1006.43; *C_v_ =* 1875.2; *C_w_ =* 4183, [J/(kg∙K)] and *λ_g_* = 0.0259; *λ_w_* = 0.612; *λ_s_* = 0.7, [W/(m∙K)]. The specific heat of the liquid-vapor phase transition is *r_v_* = 2.260·10^6^, [J/kg], and the diffusion coefficient of vapor in air is *D_va_* = 2.31 × 10^−5^, [m^2^/s].

### 3.1. Evaporation Processes

At the initial time, the material moisture content is *w_l,_*_0_ = 60 [kg/m^3^]. This value produces half of the maximum possible moisture content in the material, at which time all pores are filled with liquid. At the specified moisture content, the relative air humidity inside the material is practically equal to one. The initial material temperature is 20 [°C]. The material is placed in an air medium, its temperature is also 20 [°C], the relative humidity is *φ* = 0.6. At this point, *ρ_v,∞_* = 0.0104 [kg/m^3^].

**Figure 5:**
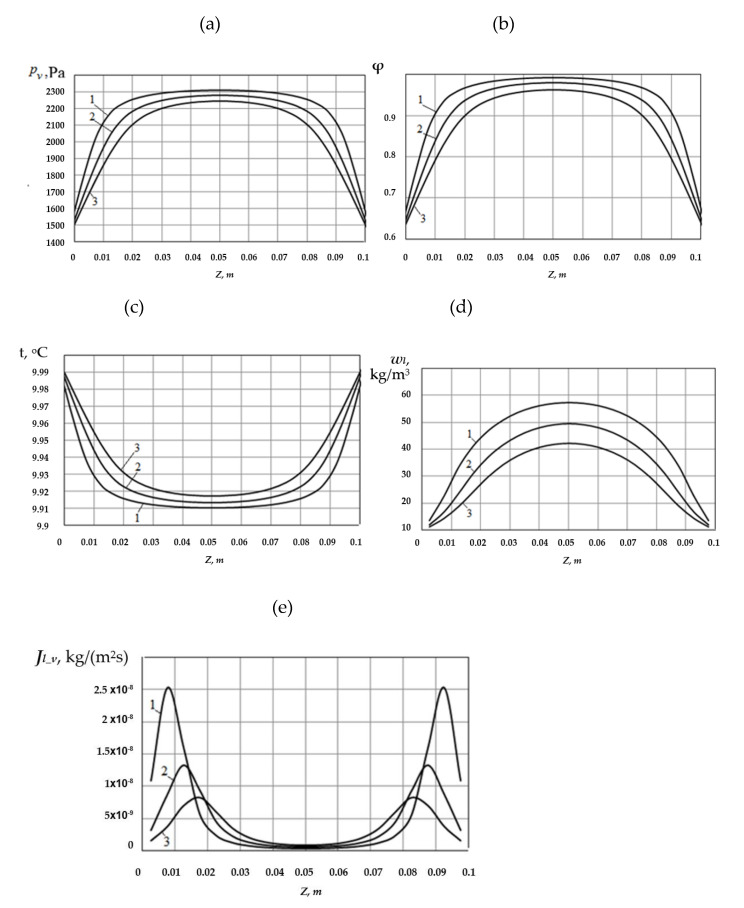
Distribution of the vapor partial pressure over the material thickness (**a**), relative air humidity (**b**), temperature (**c**), moisture content (**d**), and vapor flow density from the liquid column surfaces (**e**), moment of time: 1 − τ = 2.5 × 10^5^ s; 2 − τ = 5.0 × 10^5^ s; 3 − τ = 7.5 × 10^5^ s.

The calculation results of the variation with time in temperature and moisture conditions of the capillary-porous material for these conditions are shown in Figure 5.

As is shown in Figure 5a,b, the partial pressure of water vapor, as well as relative air humidity inside the material, decrease with time. The maximum values of these quantities are observed in the middle section of the material. In the direction of heat and mass exchange surfaces (*z* = 0 and *z* = 0.1 m), these quantities decrease to their values in the external medium. The material moisture content *w_l_* changes in a similar way to *w_l_* (Figure 5d).

As a result, at the initial time, the material and environment temperature are identical, while the material internal energy is spent on the evaporation process in the initial period of heat and mass transfer. Therefore, its temperature initially decreases and becomes lower than the initial value. Then, as the external medium temperature becomes higher than the material temperature, heat flows into the material from the outside. This heat is spent on the evaporation process and the gradual material heating. Its temperature rises over time (Figure 5c).

The distribution of evaporated vapor mass flows $J_{l\_v,dif}$ over the material thickness is shown in Figure 5e. As is shown in this figure, the evaporation process inside the material most intensely occurs in the areas near its surfaces. Over time, the maxima of curves $J_{l\_v,dif}\left(z\right)$ gradually move into the material.

In the second example, a material with the same initial parameters is placed in an air medium at 35 [°C]. The partial density of water vapor in the air medium is the same as in the first case: *ρ_v,∞_* = 0.0104 [kg/m^3^]. Naturally, the relative humidity of the external air medium falls down to *φ* = 0.26.

**Figure 6:**
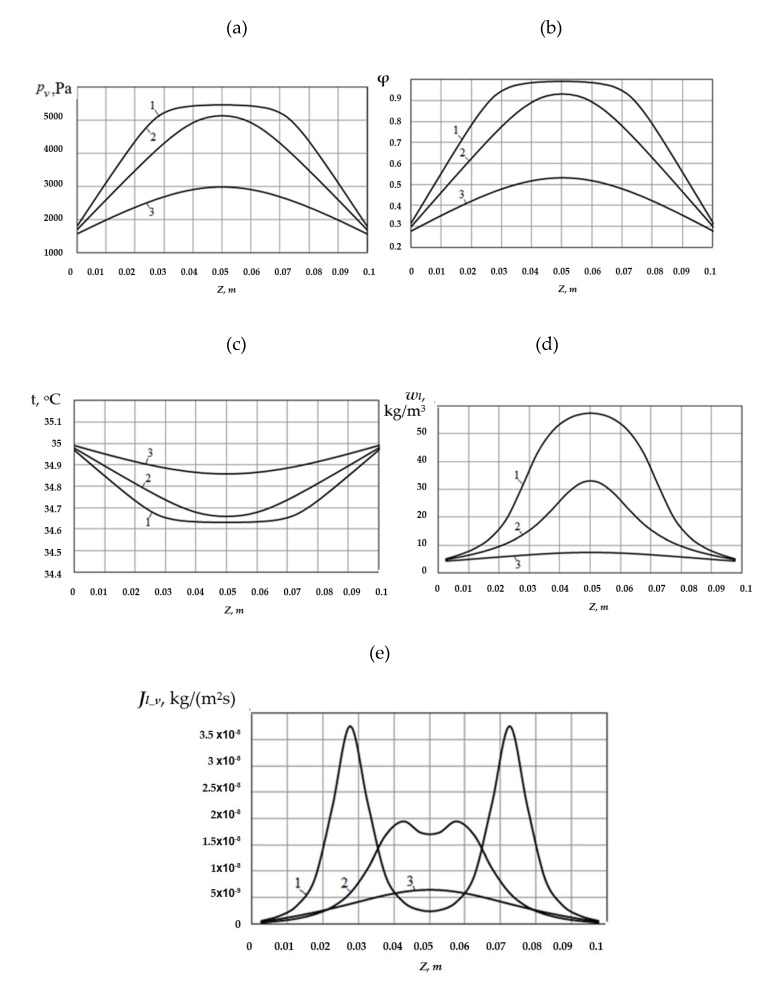
Distribution of the vapor partial pressure over the material thickness (**a**), relative air humidity (**b**), temperature (**c**), moisture content (**d**), and vapor flow density from the liquid column surfaces (**e**), moment of time: 1 − τ = 2.5 × 10^5^ [s]; 2 − τ = 5.0 × 10^5^ [s]; 3 − τ = 7.5 × 10^5^ [s]

The calculation results of the variation with time in temperature and moisture conditions of the capillary-porous material for these conditions are shown in Figure 6.

As can be seen from a comparison of Figure 6 with Figure 5, the behavior of the water vapor partial pressure with time, the relative air humidity and moisture content inside the material, is basically the same as in the previously considered case, when the initial material temperature was the same as external medium temperature. However, when the medium temperature outside the material is higher than the initial material temperature, its drying is much more intensive.

It has been proven by the moisture content degree in the material *w_l_* for the same time intervals, analyzed in the first variant. If in the first variant at *τ* = 7.5 × 10^5^ [s]*,* the maximum moisture content in the middle part of the material, with its maximum, is *w_l_* = 35.65 [kg/m^3^] (Figure 5d), then in the second variant, the specified moisture content will be *w_l_* = 7.48 [kg/m^3^] (Figure 6d).

Unlike the first variant, the temperature inside the material changes with time. From the initial time, the porous material temperature starts to increase due to the initial temperature difference between the material and the external medium. The heat, entering the material from the outside, is spent on material heating and liquid evaporation inside the material.

The distribution of evaporated vapor mass flows $J_{l\_v,dif}$ over the material thickness is shown in Figure 6e.

As in the previously analyzed variant, the evaporation process inside the material in the initial period occurs most intensely in the areas near its surfaces. Over time, the maxima of curves $J_{l\_v,dif}\left(z\right)$ gradually move into the material and merge into one maximum in its middle.

### 3.2. Condensation Processes

**Figure 7:**
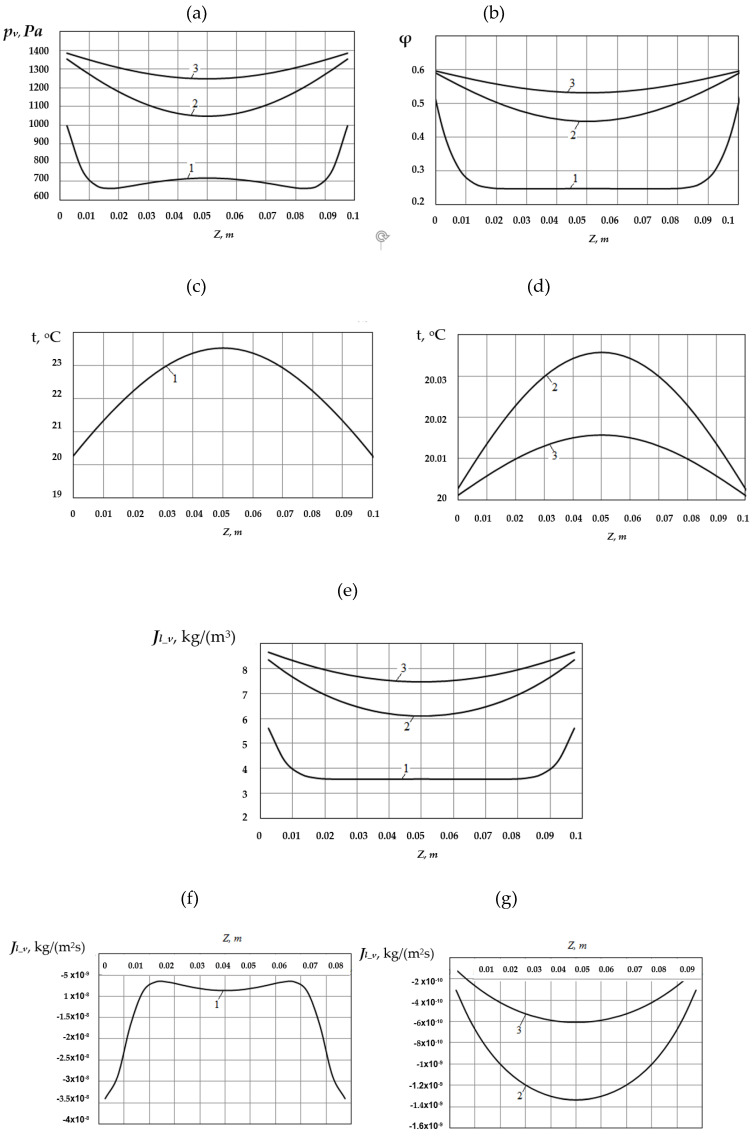
Distribution of the vapor partial pressure (**a**), relative air humidity (**b**), temperature (**c**, **d**), moisture content (**e**), and vapor flow density from the liquid column surfaces (**f**; **g**) over the material thickness and moment of time: 1 − τ = 5 × 10^3^ [s]; 2 − τ = 2.5 × 10^5^ [s]; 3 − τ = 5.0 × 10^5^ [s].

The next example considers the case when the investigated porous material, which at the initial moment of time has a temperature of *t*_0_ = 35 °C and a moisture content *w_l_*_,0_ = 3.8 [kg/m^3^], is placed in an air environment with a temperature of *t*_∞_ = 20 [°C] and a relative humidity of φ_∞_ = 0.6 In this case, as in the previous cases, ρ*_v_*_, ∞_ = 0.0104 [kg/m^3^]. Under such conditions, the process of moisture condensation begins on the surfaces of the sample, and then in its inner layers. The results of calculating the change in time of the temperature-humidity state of the sample under these conditions are shown in Figure 7.

As seen from Figure 7a,b, as a result of moisture condensation on the surfaces and inside the material, the partial pressure of water vapor, as well as the relative humidity of the air inside the material, increases over time. Over time, the moisture content of the porous material also increases (Figure 7e) and approaches the value of the maximum hygroscopic value corresponding to the humidity of the outside air. The temperature on the surfaces and inside the material gradually decreases (Figure 7c,d).

The modulus of the vapor flux density $J_{l\_v,dif}\left(z\right)$, which condenses inside the material (the flux itself is negative), has its maximum values at the surface in the initial period. Over time, the maximum value of the modulus of the vapor flow $J_{l\_v,dif}\left(z\right)$, which condenses inside the material, moves to the middle of the sample.

## 4. Discussion

As follows from the presented results, the analyzed network model of a wet capillary-porous material can be used to calculate the dynamics of changes in its temperature and moisture conditions.

This model makes it possible to calculate distribution over the thickness and change in time of the partial pressure of water vapor, temperature and liquid moisture content inside the material with the changes in temperature and moisture of the outside air.

According to the results of computational studies, evaporation (or condensation) inside the pores of a material with a change in external conditions occurs more intensively near its boundaries. Over time, the most intense areas of evaporation pass into the depth of the material. Note that the dynamics of temperature change are more intense than the dynamics of changes in humidity and moisture content.

For all considered cases, the times of establishment of thermodynamic equilibrium are rather long at more than 10 days (more than 7.5 × 10^5^ s). Such dynamics logically correspond to the physics of the resulting effects.

The model is non-equilibrium, it is based on the differences in the parameters of the state of the vapor-gas and liquid phases in the micropores of the material. The difference in temperatures reached up to 1.5 [°C], in pressures-up to 5 [MPa], mainly due to the capillary pressure in the microchannel with a solid liquid. By the way, the capillary pressure was not specified by an analytical expression containing the surface tension, but the original tabular data [9] were used, taking into account the deviation of the microchannel from a strict cylindrical shape, for example, its possible cone shape.

The model is not devoid of limitations and incompleteness of taking into account the accompanying physical effects. It is applicable for a temperature range of no less than 0 [°C] and no more than 100 [°C]. At subzero temperatures, water freezes (or ice melts), and such a phase transformation is not taken into account. At temperatures above 100 [°C], it is necessary to complicate the model and take into account the effects of volumetric boiling of the liquid. The model also does not take into account the adhesion of vapor molecules on the surface of the solid material of the walls of the microchannel, does not take into account the possible film flow over the surface of the walls and does not take into account possible structural modes of liquid flow in the microchannel, such as slug, foam, dispersed and other flows.

Possible further studies of the proposed model are as follows. First of all, this could involve checking the model for: sensitivity to changes in fixed parameters and characteristics of a solid material (pore diameter, integral porosity index, its permeability); dependence of thermophysical characteristics on temperature and pressure; the structure of the filtration fluid flow; and other factors. It is of interest to make similar calculations for other materials, for example, thermal insulation. It is possible to develop a model for a different pore shape, for example, a spherical one.

It is extremely interesting to compare the model calculations with some data that were previously obtained by the authors in the experimental study of the dynamics of changes in the moisture content of a number of building and heat-insulating materials, depending on the humidity of the surrounding air.

In studying the condensation processes, calculations showed that in the first 5 min the specific mass flows of water vapor are very high—they reach 10^−6^ [kg/(m^2^∙s)] and higher. It can result in the formation of a continuous film of dropping liquid (water) on the material (brick) surface and complete filling of pores with water in a thin near-surface layer to a material depth of 0.5...1 [mm]. When the ambient temperature drops to sub-zero values (in degrees Celsius), this condition can result in the ice formation in near-surface micropores. A further decrease in temperature is accompanied by volumetric expansion of ice, leading to microdestructions of the material surface, leading to a loss of surface strength. Therefore, the modeling results of water vapor condensation can be applied to engineering calculations in the processes and technologies against the surface microdestruction in facade building structures.

## 5. Conclusions

The presented model includes a certain number of parameters, thermophysical properties and characteristics of a porous material. Some of them depend heavily on moisture content. It refers chiefly to capillary pressure and filtration coefficients. The computational model also describes the dependence of the material equilibrium moisture content on the air relative humidity (sorption curve). To provide solid results on the temperature-moisture condition of a porous material, based on the proposed calculation model, reliable data on the specified characteristics of materials are required. These characteristics for specific materials should be obtained from complex experimental studies using special laboratory facilities, which is a research problem to be solved. To derive required thermophysical characteristics of the studied material from the experimental data, we may use the proposed transfer model to solve inverse problems of heat and mass transfer.

It is also important to obtain reliable information on the structure of porous materials based on modern optical or electronic microscopy, using fluorescent substances that fill the pores.

The developed model can be effectively used in describing the processes of drying capillary-porous materials; in fact, from the problem involving this area of heat and mass transfer, the original problem statement arose. This model is probably not quite suitable for studying colloidal structures.

It is also advisable to thoroughly check the model (verification or validation) using other numerical modeling approaches, for example, using the LBM model or direct CFD-modeling.

Undoubtedly, such bifurcations of the further use of the model will require its corresponding correction, adjustment and, of course, time.

## Data Availability

The data presented in this study is available upon request from the respective author. The article presents a new mathematical model and calculation method that are available for use. A computer program may be available from the authors of the article.
